# Antiproliferative effect of indeno[1,2-d]thiazolo[3,2-a]pyrimidine analogues on IL-6 mediated STAT3 and role of the apoptotic pathway in albino Wistar rats of ethyl carbamate-induced lung carcinoma: In-silico, In-vitro, and In-vivo study

**DOI:** 10.1186/s12935-024-03390-6

**Published:** 2024-06-26

**Authors:** Archana Bharti Sonkar, Abhishek Verma, Sneha Yadav, Rohit Kumar, Jyoti Singh, Amit K. Keshari, Soniya Rani, Anurag Kumar, Dharmendra Kumar, Neeraj Kumar Shrivastava, Shubham Rastogi, Mariam K. Alamoudi, Mohd Nazam Ansari, Abdulaziz S. Saeedan, Gaurav Kaithwas, Sudipta Saha

**Affiliations:** 1 Department of Pharmaceutical Sciences, School of Pharmaceutical Sciences, Babasaheb Bhimrao Ambedkar University (A Central University), Vidya Vihar, Raebareli Road, Lucknow, Uttar Pradesh 226 025 India; 2 Amity Institute of Pharmacy, Amity University, Lucknow campus, Lucknow, Uttar Pradesh 226028 India; 3 Department of Pharmacology, College of Pharmacy, Prince Sattam Bin Abdulaziz University, Al-Kharj, Saudi Arabia

**Keywords:** Lung cancer, Thiazolo[3,2-a]pyrimidine, Urethane, Anti-inflammatory, Anti-proliferative, Ethyl carbamate

## Abstract

Lung cancer (LC) ranks second most prevalent cancer in females after breast cancer and second in males after prostate cancer. Based on the GLOBOCAN 2020 report, India represented 5.9% of LC cases and 8.1% of deaths caused by the disease. Several clinical studies have shown that LC occurs because of biological and morphological abnormalities and the involvement of altered level of antioxidants, cytokines, and apoptotic markers. In the present study, we explored the antiproliferative activity of indeno[1,2-d]thiazolo[3,2-a]pyrimidine analogues against LC using in-vitro, in-silico, and in-vivo models. In-vitro screening against A549 cells revealed compounds 9B (8-methoxy-5-(3,4,5-trimethoxyphenyl)-5,6-dihydroindeno[1,2-d]thiazolo[3,2-a]pyrimidine) and 12B (5-(4-chlorophenyl)-5,6-dihydroindeno[1,2-d]thiazolo[3,2-a]pyrimidine) as potential pyrimidine analogues against LC. Compounds 9B and 12B were docked with different molecular targets IL-6, Cyt-C, Caspase9, and Caspase3 using AutoDock Vina 4.1 to evaluate the binding affinity. Subsequently, in-vivo studies were conducted in albino Wistar rats through ethyl-carbamate (EC)- induced LC. 9B and 12B imparted significant effects on physiological (weight variation), and biochemical (anti-oxidant [TBAR’s, SOD, ProC, and GSH), lipid (TC, TG, LDL, VLDL, and HDL)], and cytokine (IL-2, IL-6, IL-10, and IL-1β) markers in EC-induced LC in albino Wistar rats. Morphological examination (SEM and H&E) and western blotting (IL-6, STAT3, Cyt-C, BAX, Bcl-2, Caspase3, and caspase9) showed that compounds 9B and 12B had antiproliferative effects. Accordingly, from the in-vitro, in-silico, and in-vivo experimental findings, we concluded that 9B and 12B have significant antiproliferative potential and are potential candidates for further evaluation to meet the requirements of investigation of new drug application.

**Supplementary Information:**

The online version contains supplementary material available at 10.1186/s12935-024-03390-6.

## Introduction

Lung cancer (LC) is the major cause of cancer incidence and mortality [1] and is the second leading cancer in males and females [2]. In India, LC accounts for ∼ 5.9% (in females) and ∼ 8.5% (in males) of all cancer-related deaths [3, 4]. Males have a higher incidence and mortality than females globally [5, 6]. Therefore, identifying or synthesizing new chemical moieties that target molecular pathways is a key step in the development of new treatment approaches. Researchers have reported that thiazolo[3,2-a]pyrimidine is a group of heterocyclic compounds known to have various significant biological properties such as antimicrobial, antipsychotic, anti-inflammatory, anti-parkinson’s, antidepressant, and anti-HIV [7, 8].

These molecules have increased the considerable scope of the anticancer activity of thiazolo[3,2-a]pyrimidine-related compounds. Since the last decade, broad-spectrum standard anti-cancerous drugs such as camptothecin, doxorubicin, paclitaxel, and 5-fluorouracil (5-FU) have been extensively used for multiple treatments. 5-FU or 5-fluro-2, 4-pyrimidinedione is a chemotherapeutic drug frequently delivered either by a single medication or in combination with other therapeutic regimens [9–12]. 5-FU is an anticancer antimetabolite drug primarily derived from thiazolo-pyrimidines that comprise a thiazole and a pyrimidine ring, which inhibit the proliferation of cancer cells [13, 14]. This has led to considerable hope for the development of new chemical moieties with pyrimidine ring.

Thiazolopyrimidine ring systems possess antitumor activity. The previously reported significance of such synthons has generated interest in exploiting this valuable structure in the design and synthesis of new thiazolo-pyrimidines analogues as antitumor agents [15].

Moreover, a series of these molecules had already been synthesized in our laboratory [16]. The structures of the synthesized products were confirmed using spectroscopic techniques such as mass and NMR spectrometry [16].

Therefore, in continuation of our previous work, this study has been designed to explore the anticancer potential of the synthesized thiazolo[3,2-a]pyrimidine analogues (9B (8-methoxy-5-(3,4,5-trimethoxyphenyl)-5,6-dihydroindeno[1,2-d]thiazolo[3,2-a]pyrimidine), Mol. wt. 422.50 and 12B (5-(4-chlorophenyl)-5,6-dihydroindeno[1,2-d]thiazolo[3,2-a]pyrimidine), Mol. wt. 336.84) against LC.

## Materials and methods

### Drugs and reagents

EC was procured from the Tokyo chemical industry, Japan. IL-2 (RAB0288) and IL-6 (RAB0311) ELISA kits were purchased from Sigma-Aldrich, Bengaluru, India. IL-1β (GX3930E1) and IL-10 (GX8140E1) experimental ELISA kits were obtained from Genetix Biotech Asia Pvt. Ltd, India. Caspase-9 (ITER0804) and Caspase-3 (KHO1091) ELISA kits were purchased from Invitrogen and Thermo Fisher, India, respectively. All primary antibodies, including IL-6 (SC-32,296), STAT-3 (SC8019), BAX (SC23959), Bcl-2 (SC7382), Cyt-C (SC13561), Caspase-9 (SC73548), Caspase-3 (SC56052), β-actin (SC517582), were procured from Santa Cruz Biotechnology, USA. All other remaining chemicals and solvents of molecular grade were purchased from Hi-Media laboratories, Sigma-Aldrich, India.

### In-vitro cell cytotoxicity assay

To determine the cytotoxicity potential of the synthesized compounds. All samples were sent to the ACTREC, Tata Memorial Centre, Mumbai, India. They followed the standard protocol for sample analysis of the synthesized compounds. Adriamycin (ADR) was selected as a standard at 10, 20, 40, and 80 µg/mL to screen for indeno [1, 2-d] thiazolo [3,2-a] pyrimidine analogues. GI50 growth inhibition of 50% was calculated using the [(Ti − Tz)/(C − Tz)] ×100% formula, where C, controlled growth; Tz, time zero growth; and Ti, test growth at four concentration levels in the presence of the drug [17]. The GI50 value of the compounds was considered to demonstrate antiproliferative activity against A549 cells [18–20].

### In-silico molecular docking analysis

Docking study of the synthesized compounds were performed using IL-6, Cytochrome-C (Cyt-C), Caspase 9, and Caspase 3. The molecular structure of the ligands 9B and 12B was generated using ChemDraw Profession 16.0. Additionally, we utilized the NCBI (https://www.ncbi.nlm.nih.gov/protein) and RCSB (https://www.rcsb.org) databases as sources to obtain the desired protein targets: IL-6 (1ALU), Cyt-C (5TY3), caspase 9 (2AR9), and caspase 3 (2XYG). 3D structures of different target proteins were prepared in PDB format [21] using AutoDock Vina 4.1. DS visualizing software (Discovery Studio Visualizer, 2021) was used to remove specific protein targets, water molecules [22], hetatms (hetero atoms) from the co-crystal structure, and active pocket sites of protein by utilizing the “Active site determination from PDB site record” function. To dock the ligand with desire proteins (kcal/mol), we used AutoDock Vina 4.1 [22–25]. AutoDock Vina 4.1 [25] calculated the predicted binding energy and expressed it in kcal/mol [26]. The interaction between these complexes was analyzed using the DS visualizer, and the potential hydrogen bonds, hydrophobic amino acid interactions in close proximity were also examined.

### Acute toxicity study

It is necessary to investigate the safety and efficacy of the synthesized compounds in vivo, which was performed as per the guidelines of the Organization for Economic Cooperation and Development guidelines 423 (OECD 423). The synthesized compounds were orally administered at doses of 5, 10, and 15 mg/kg body weight to albino Wistar rats for 15 days (*n* = 5), and the animals were observed every day for any toxic signs at various doses. Morphological and oxidative stress parameters were used to determine dose-related toxicity.

### Pharmacokinetic study using high-performance liquid chromatography (HPLC)

9B and 12B compounds were orally administered at 10 mg/kg (p.o. dose obtained from acute toxicity studies) to rats, and blood was collected from the latetal tail vein at 0.083, 0.25, 0.5, 1, 2, 4, 8, 12, 16, 24, and 48 h [27]. After collection, blood was centrifuged and plasma was separated, which was kept at -20 °C for further HPLC determination. Chromatographic separations were performed using a Shimadzu Prominence-I LC-2030 Plus equipped with HPLC and a photodiode array (PDA) detector. The mobile phase consisted of a gradient elution of 2:8, water: methanol, and a continuous flow rate of 1 mL/min in an RP-C18 column (5.0 µ particle sizes, 4.6 mm internal diameter, and 250 mm length) with different λmax of 310 and 320 nm, respectively, at 40 °C throughout the experiment. The column was washed using an elution solvent (water: methanol = 50:50) after each run, and the final output was analyzed using WinNonlin version 1.5.3 software.

### Animal and study design

Six-week-old experimental male albino Wistar rats (100–120 g) were procured from the animal house facility of Babu Banarasi Das Northern India Institute of Technology Lucknow and approved by the Institutional Animal Ethical Committee (CPCSEA No. 809/PO/Re/S/03/CPCSEA) with approval no. BBDNIIT/IAEC/APR/2022/06. The animal welfare protocol and experiments were performed as per the CPCSEA guidelines for laboratory animals and ethics, Department of Animal Welfare, Government of India. They were randomly distributed into five groups of eight animals each. The experimental rats were acclimated under standard research laboratory conditions with free access to standard rat chow and water *ad libitum*. Group 1 (NC, normal control group): 0.25% CMC (2 mL/kg, p. o.), Group 2 (CC, carcinogen control/EC-exposed group): EC (0.375 mg/kg, four i.p. injections within a gap of three weeks between each dose over twelve weeks period), Group 3 (PC, positive control group): EC + 5-FU (10 mg/kg, i.p. for 15 days after EC induced LC), Group 4 (9B): EC + Compound 9B (10 mg/kg, p.o. for 15 days after EC induced LC), and Group 5 (12B): EC + Compound 12B (10 mg/kg, p.o. for 15 days after the induction of LC). At the end of the treatment, the animals were euthanized by cervical decapitation after anesthesia using a combination of diazepam and ketamine hydrochloride (5 mg/kg, and 100 mg/kg, i.m. respectively). The lung was excised immediately, rinsed in ice-cold saline, and stored at -80 °C for further study. Serum was collected and stored for further analysis.

## In-vivo antineoplastic studies of 9B and 12B

### Estimation of the various physiological parameters

#### Weight variation

Change in body weight (gm) was measured on the initial and final day of the experiment, and percent (%) weight gain/loss was calculated.

### Measurement of the serum lipid profile

Serum total cholesterol (TC), triglycerides (TG), and high-density lipoprotein (HDL) levels were measured by using lipid profile kits (Agappe Diagnostic Ltd., Kerala, India). In brief, the assay was performed according to the manufacturer’s instructions. Low-density lipoprotein (LDL) and very-low-density lipoprotein (VLDL) levels were estimated and calculated using Friedewald’s formula [24].

### Measurement of the oxidative changes

Biochemical parameters such as protein carbonyl (PC), superoxide dismutase (SOD), glutathione (GSH), and thiobarbituric acid reactive substances (TBARs) were estimated in 10% lung tissue homogenate using our previously standardized laboratory protocols [17, 28, 29].

### Cytokine estimation

The pro-inflammatory cytokines IL-2, IL-6, IL-10, and IL-1β were assayed using commercially available kits according to the manufacturer’s instructions.

### Histopathological analysis

Histopathological studies were conducted to determine intracellular changes in lung tissue. Tissue samples were stained with H&E (haemotoxyline and eosin) using a methodology established previously at our laboratory for histological analysis and observed under an optical microscope (Steindroff N-120, 40X).

### Scanning electron microscopy analysis

Lung tissue samples of 2–4 mm thickness were sectioned, and the samples were prepared by fixation, washing, post-fixation, and dehydration according to the standardized procedures of our laboratory. Finally, the samples were mounted on aluminium stubs with adhesive tape and examined for morphological changes using SEM (JEOL JSM-6490LV).

### Western blot analysis

Protein expression levels of IL-6 (pro-inflammatory), STAT-3 (pro-inflammatory), Bax (pro-apoptotic), Bcl-2 (anti-apoptotic), Cyt-C (pro-apoptotic), Caspase-9, and Caspase-3 (pro-apoptotic/executor caspase) were assessed by immunoblotting. Proteins were electrophoresed on sodium dodecyl sulphate polyacrylamide gel (SDS-PAGE) and probed to detect molecules of interest in the mixture. The membrane was developed with enhanced chemiluminescence ECL, and images were obtained using Chemidoc XRS+ (Bio-rad).

## Result

### In-vitro cell cytotoxicity assay

**Fig. 1:**
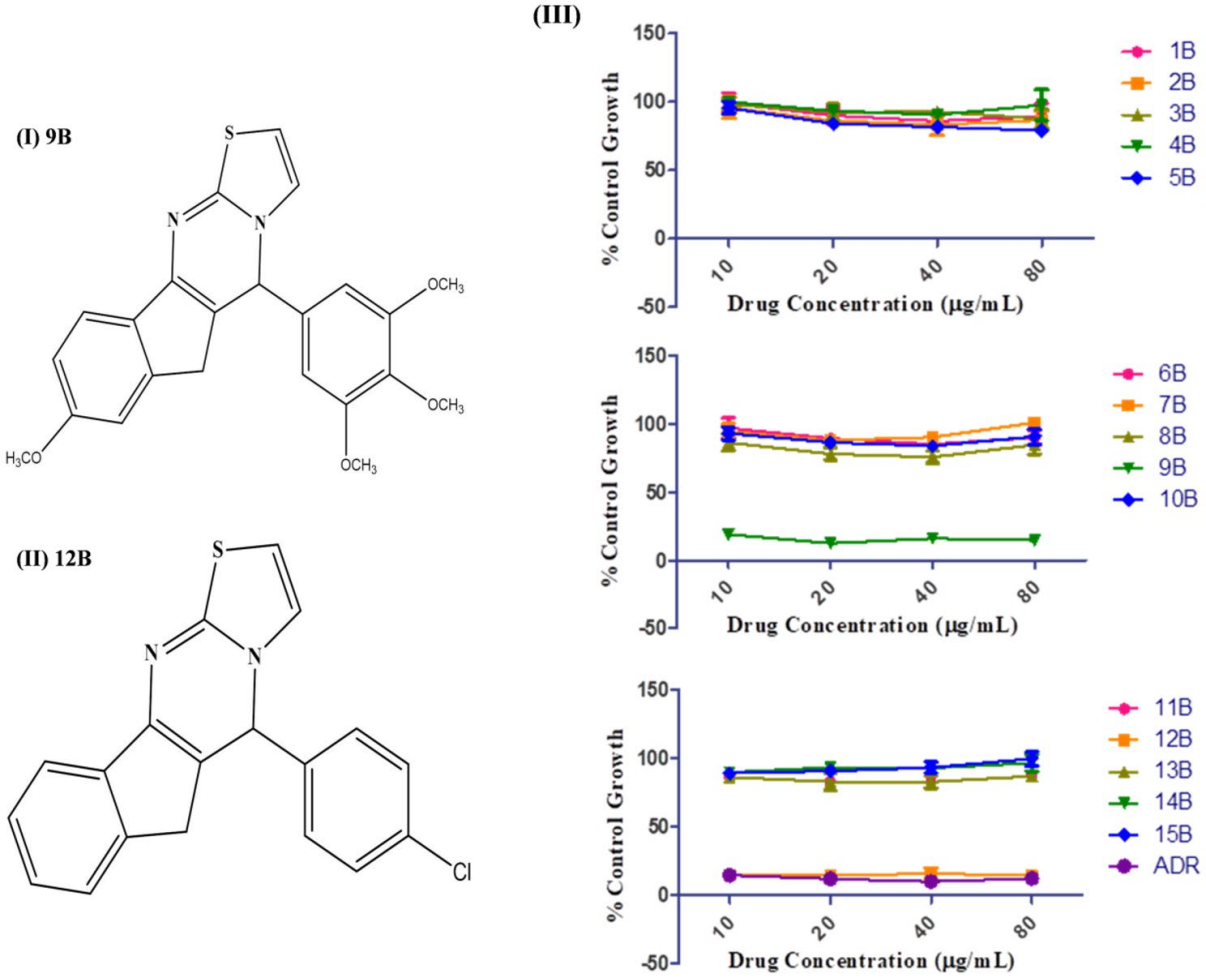
**(I)** Structure of 9B (8-methoxy-5-(3,4,5-tri-methoxyphenyl)-5,6-dihydroindeno[1,2-d]thiazolo[3,2-a]pyrimidine), **(II)** 12B (5-(4-chlorophenyl)-5,6-dihydroindeno[1,2-d]thiazolo[3,2-a]pyrimidine), and **(III)** SRB assay of (1B-15B and ADR) on human lung cancer cell line A549, graph plot between drug concentration (µg/ml) vs. % control growth

Thiazolo [3, 2-a] pyrimidine-containing compound series (1B-15B) were analzsed against adriamycin (ADR) using the A549 cell line for cytotoxic estimation, as depicted in Fig. 1. Based on the GI50 value of the series, 9B (< 20) and 12B (< 10) were found to be the most potential compounds (Fig. 1 (III)). Consequently, 9B and 12B were considered for further evaluation.

### In-silico molecular docking analysis

**Fig. 2:**
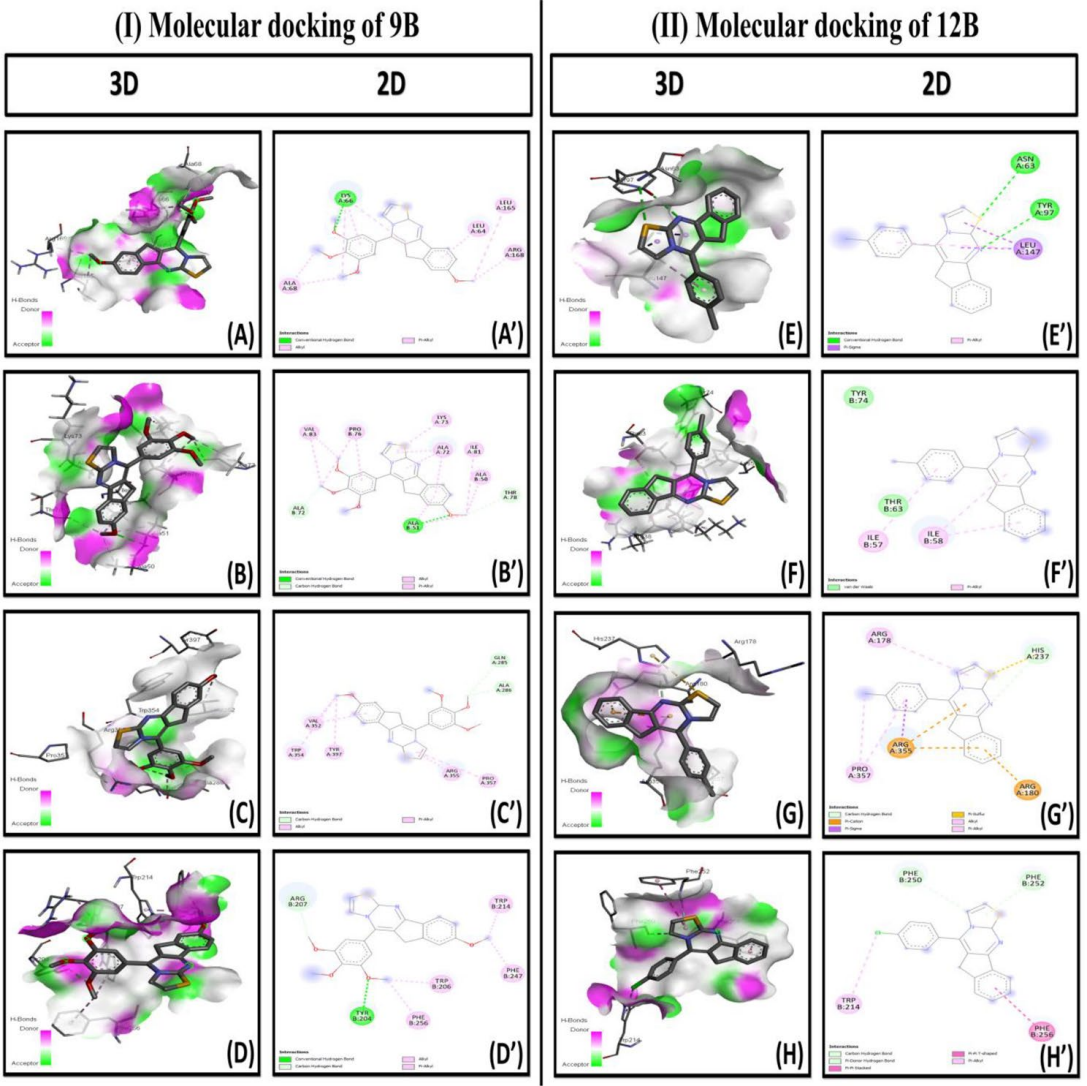
Molecular docking of 9B and 12B. **(I)** 3D and 2D molecular docking images of 9B with (A and A’) IL-6 (PDB: 1ALU), (B and B’) Cyt-C (PDB: 5TY3), (C and C’) Caspase 9 (PDB: 2AR9) and (D and D’) Caspase 3 (PDB: 2XYG) and **(II)** 3D and 2D docking images of 12B with (E and E’) IL-6 (PDB: 1ALU), (F and F’) Cyt-C (PDB: 5TY3), (G and G’) Caspase 9 (PDB: 2AR9) and (H and H’) Caspase 3 (PDB: 2XYG). Comparative studies were performed using AutoDock Vina 4.1

We performed docking studies of the synthesized compounds with the molecular targets IL-6 (PDB: 1ALU), Cyt-C (PDB: 5TY3), Caspase 9 (PDB: 2AR9), and Caspase 3 (PDB: 2XYG) using AutoDock Vina 4.1. The binding affinities (kcal/mole) and the number of probable hydrogen bonds was evaluated. The binding affinities for 9B (-6.1, -6.3, -7.4, and − 7.2), and 12B (-7.0, -6.2, -7.5, and − 7.3) were found to be with IL-6, Cyt-C, Caspase 9, and Caspase 3, respectively (Fig. 2 (I and II)) which indicates the amino-acid interactions with both ligands. Hydrongen bonds involvements with 9B and 12B were showed in Table 1.

**Table 1:** Binding interaction of compound 9B and 12B with IL-6 (PDB: 1ALU), Cyt-C (PDB: 5TY3), Caspase 9 (PDB: 2AR9), and Caspase 3 (PDB: 2XYG). Comparative studies were performed using AutoDock Vina 4.1

Lead molecule (Ligand)	Target Protein (PDB ID)	Amino acids are involved in hydrophobic interactions	Residues involved in Hydrogen bonds	Affinity (kcal/mol)
9B	IL-6 (1ALU)	LYS A:66, LEU A:64, LEU A:165, ARG A:168, ALA A:68	LYS A:66	-6.1
	Cyt-C (5TY3)	VAL A:83, PRO B:76, LYS A:73, ALA A:72, ILE A:81, ALA B:50, THR A:78, ALA B:51, ALA B:72	ALA B:51, THR A:78, ALA B:72	-6.3
	Caspase 9 (2AR9)	VAL A:352, GLN A:285, ALA A:286, PRO A:357, ARG A:355, TYR A:397, TRP A:354	GLN A:285, ALA A:286	-7.4
	Caspase 3 (2XYG)	ARG B:207, TRP B:214, PHE B:247, TRP B:206, PHE B:256, TYR B:204	ARG B:207, TYR:204	-7.2
12B	IL-6 (1ALU)	ASN A:63, TYR A: 97, LEU A:147	ASN A:63, TYR A:97	-7.0
	Cyt-C (5TY3)	THR B:63, ILE B:58, ILE B:57	-	-6.2
	Caspase 9 (2AR9)	ARG A:178, HIS A:237, ARG A:180, ARG A:355, PRO A:357	HIS A: 237	-7.5
	Caspase 3 (2XYG)	PHE B: 250, PHE B:252, PHE B:256, TRP B:214	PHE B:250, PHE B:252	-7.3

### Acute toxicity study

Both compounds were orally administrated (9B and 12B) for 15 days at different doses, such as 5, 10, and 15 mg/kg, and showed no toxic effect. No mortality was observed in the animals during this study. Histological examination of lung, liver, and kidney tissue, we could not find any toxic effect. Moreover, antioxidant marker estimation showed no change in SOD, CAT, GSH, and TBARs values. Based on our above findings, we proceeded with a 10 mg/kg dose, which is similar to 5-FU for *in-vivo* anti-proliferative activity evaluation (Supplementary Figs. 1 and 2).

### Pharmacokinetic study

**Fig. 3:**
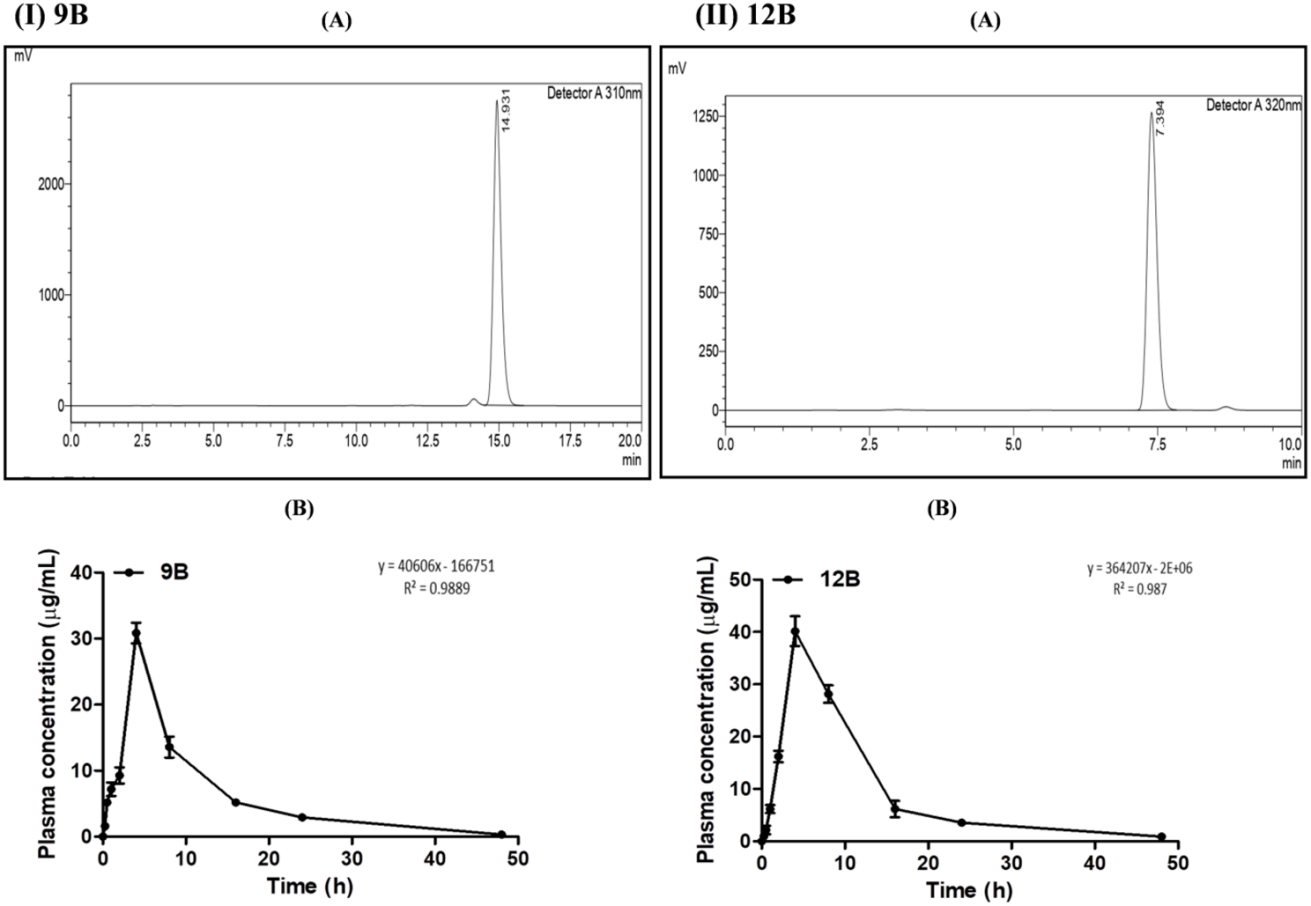
A pharmacokinetic study of 9B and 12B using HPLC; **(I)** Pharmacokinetic profiling of 9B (A) Chromatogram of 9B (RT 14.931 min) through HPLC, (B) Plasma drug concentrations at various time points after oral administration of 9B in albino Wistar rats **(II)** Pharmacokinetic profiling of 12B (A) Chromatogram of 12B (RT 7.394 min) through HPLC, (B) Plasma drug concentrations at various time points after oral administration of 12B in albino Wistar rats

Pharmacokinetic profiling of 9B and 12B at 10 mg/kg was evaluated using HPLC. 1 µg/ml − 100 µg/ml gave a 0.9889 correlation coefficient for 9B and 0.987 for 12B. The retention time of 9B was 14.931 and that of 12B was 7.394. The plasma concentration-time profiles of 9B and 12B were analyzed by using GraphPad Prism 5.02. 12B had a higher plasma distribution compared with 9B at the same time (Fig. 3 (I) 9B (A, and B) and (II) 12B (A, and B)). The T_1/2_- 7.95, 11.49; T_max_ (h): 4.00, 4.00; C_max_ (µg/ mL)- 30.84 ± 1.59, 40.15 ± 2.06; AUMC (µg.h^2^/ mL)- 3028.82, 4557.60; MRT (h)- 10.59 ± 0.99, 10.44 ± 1.08; CL (h)- 0.003, 0.006; AUC_0−∞_ (µg.h/mL)- 287.60, 436.49 for 9B and 12B, respectively. 12B showed a good biological availability as compared to 9B (Table 2).

**Table 2:** Pharmacokinetic study of 9B and 12B in rat plasma

Parameter	9B	12B
T_1/2_	7.95	11.49
T_max_(h)	4.00	4.00
C_max_ (µg/ mL)	30.84 ± 1.59	40.15 ± 2.06
AUMC (µg.h^2^/ mL)	3028.82	4557.60
MRT (h)	10.59 ± 0.99	10.44 ± 1.08
CL (h)	0.003	0.006
AUC_0−∞_ (µg.h/mL)	287.60	436.49 ± 8.59

*Abbreviations* T1/2, the time required to reach 50% plasma concentration; Tmax, the time required to reach the maximum plasma concentration; *C*max, maximum plasma concentration; AUMC, the area under the first-moment curve; MRT, mean residence time; CL, clearance; AUC, the area under the curve

## In-vivo antineoplastic studies of 9B and 12B

### Estimation of the various physiological parameters

#### Weight variation

**Fig. 4:**
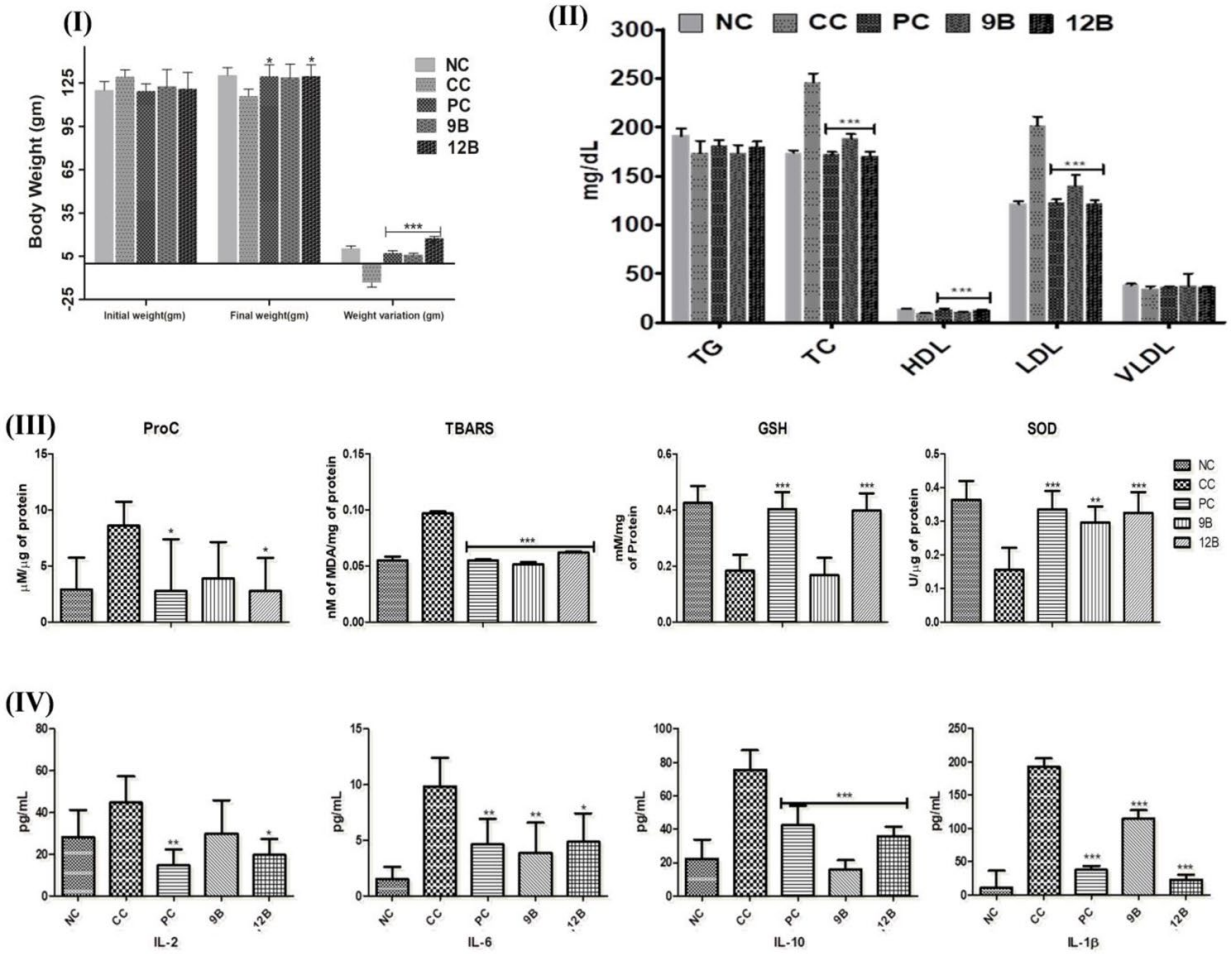
Physiological and biochemical changes in EC-induced lung carcinogenic rats after oral administration of 9B and 12B at 10 mg/kg for 15 days. **(I)** Changes in body weight of experimental animals, **(II)** Effects of 9B and 12B, on lipid profile including TG, TC, HDL, LDL, and VLDL. **(III)** Effects of 9B and 12B, on oxidative stress estimation of SOD (superoxide dismutase), PC (Protein carbonyls), TBARS (Thiobarbituric acid reactive substances), and GSH (Glutathione), and **(IV)** Effects of 9B and 12B, on pro-inflammatory cytokines such as IL-2, IL-6, IL-10, and IL-1β. Each value is represented here as mean ± SD (*n* = 6/group), statistically significant differences were observed between lung carcinogenic (CC) and test groups (9B and 12B) [one-way ANOVA followed by Bonferroni multiple comparison tests for biochemical estimation, statistical significance differences were considered concerning control (***p < 0.001, **p < 0.01 and *p < 0.05)]. Where, NC: Normal Control, CC: Carcinogen Control, PC: Positive Control, 9B (10 mg/kg), 12B (10 mg/kg)

Weight variation in rats against EC showed significant weight loss (-13.31 ± 3.15 gm). NC, PC, 9B, and 12B rats exhibited increased weight compared with the CC group (10.42 ± 1.72, 7.21 ± 1.75, 6.01 ± 1.08, and 17.52 ± 1.33gm, respectively) (Fig. 4 (I)).

### Measurement of the serum lipid profile

Figure 4 (II) indicates the cancer risk associated with the administration of EC, which further increased the levels of TC, TG, and LDL as well as the decreased levels of HDL and VLDL in the CC group. The EC-exposed group was more associated with a significant increase in TC and LDL levels, as well as a decrease in HDL levels (Fig. 4 (II)). 9B and 12B restored the alteration of the lipid profile in the treatment groups.

### Measurement of the oxidative changes

The antioxidant potential of both 9B and 12B was estimated using the findings of SOD, GSH, ProC, and TBARS. Decreased levels of GSH and SOD and increased levels of ProC and TBARS exposed the carcinogenic effect of EC on experimental animals. From the therapeutic point of view, both compounds support the antiproliferative potential by restoring the altered levels of SOD, GSH, ProC, and TBARs (Fig. 4 (III)).

### Cytokine estimation

Increased levels of cytokine IL-1β, IL-2, IL-6, and IL-10 were significantly decreased after treatment with 9B and 12B, as depicted in Fig. 4 (IV).

#### Histopathological analysis

**Fig. 5:**
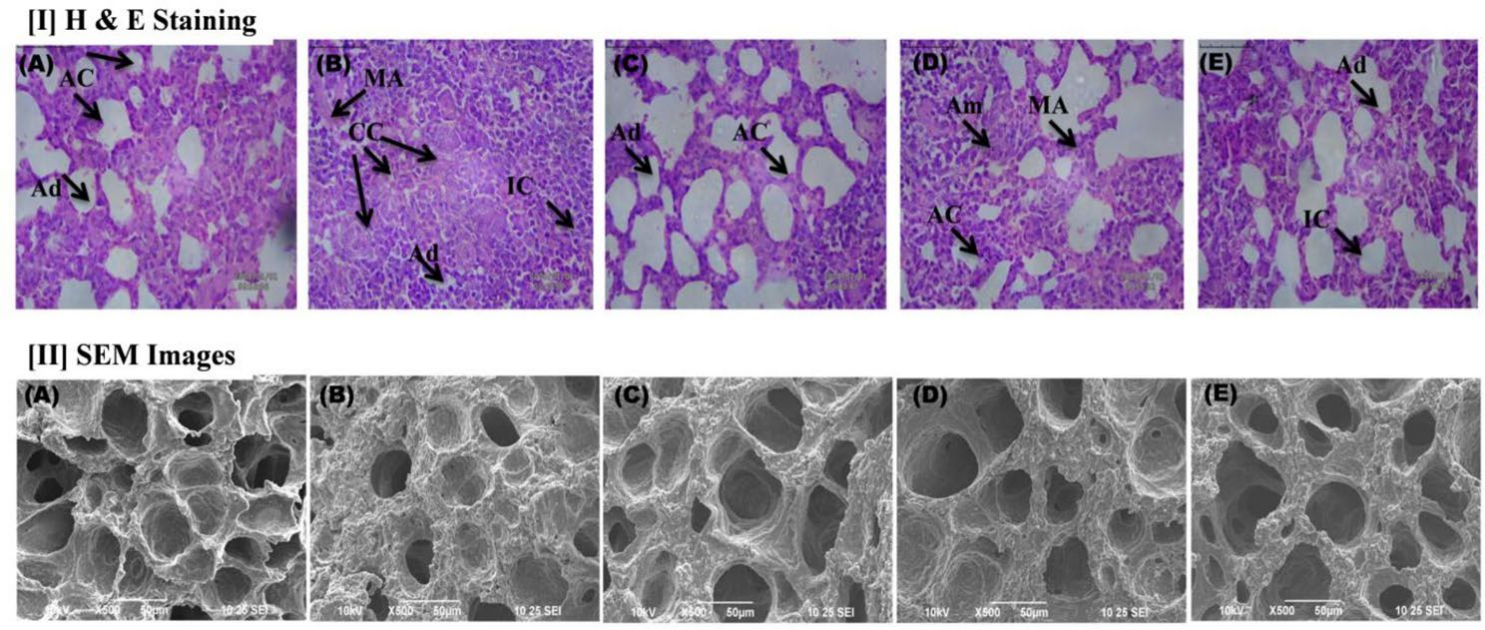
Morphological changes evaluation in EC-induced LC. **[I]** Histopathological studies of lung tissue sections of albino Wistar rats, each group analyzed under a light microscope (40X, scale bar 50 μm). Abbreviation: AC- Alveolar cell, Ad- Alveolar duct, IC- Inflammatory cell, Am- Alveolar macrophages, MA- Mucinous adenocarcinoma, CC- Carcinoma cells where (A) Normal Control [NC]; (B) Carcinogen Control [CC]; (C) Positive Control [PC]; (D) 9B; (E) 12B **[II]** Scanning electron microscopic photomicrography of lung tissue (1000X) in albino Wistar rats of each group, where (A) Normal Control [NC]; (B) Carcinogen Control [CC]; (C) Positive Control [PC]; (D) 9B; (E) 12B

In histological analysis, the number of carcinogenic nodule formations in the lungs along with structural differences, blockage of alveoli, and reduced size of alveoli confirmed the carcinogenic effect of EC. The EC effect was found to be more prominent in the CC group, which was further reduced in different groups after the treatments. The histopathological recovery of lung tissue architectures explored the therapeutic effects of 9B and 12B along with 5-FU, as shown in Fig. 5 [I].

### SEM analysis

In SEM, NC animals represent uniform tissue structure with the presence of alveolar sacks, which were perforated through alveolar ducts to provide air supply to the lungs. Pentagonal-shaped annuli containing alveolar sacs were reduced after EC administration in CC animals, and recovered after treatment with thiazolo[3,2-a]pyrimidine-containing compounds (9B and 12B), as shown in Fig. 5 [II].

#### Western blot analysis

**Fig. 6:**
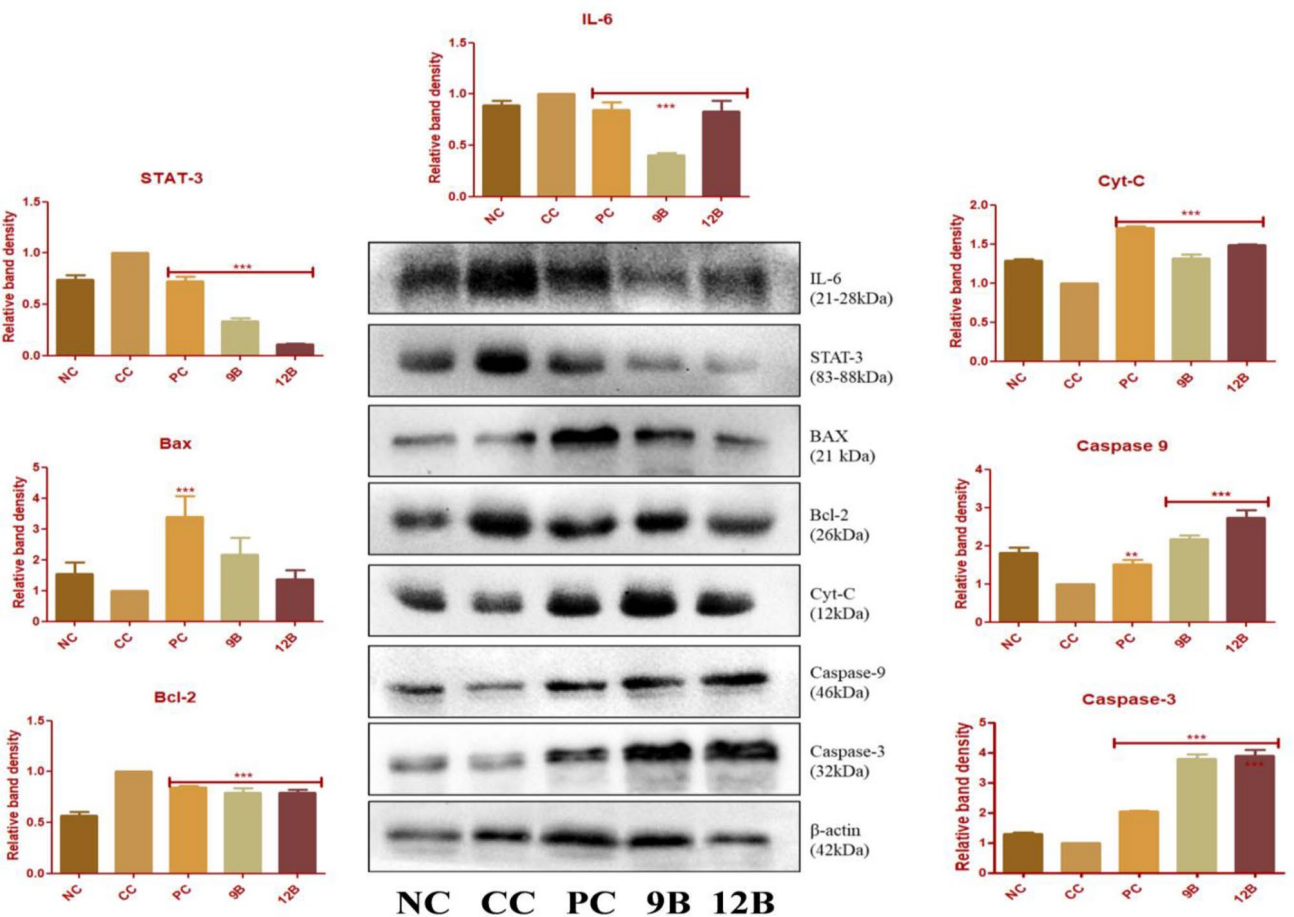
Immunoblotting assay for protein expression in lung samples; Protein expression levels of IL-6, STAT-3, Bax, Bcl-2, Cyt-C, Caspase-9, and Caspase-3 after treatment with 9B and 12B and relative band density of NC, CC, PC, 9B, and 12B with different interests of protein. Data are represented as mean ± SD. Statistically significant differences were observed between CC and treatment groups (PC, 9B, and 12B) using one-way-ANOVA followed by Bonferroni multiple comparison test (***p < 0.001, **p < 0.01, *p < 0.05)]

Administration of EC enhanced the expression of markers that promote inflammation and prevent apoptosis (IL-6, STAT-3, and Bcl-2). In addition, EC decreased the expression of pro-apoptotic markers (Bax and Cyt-c) and caspases (Caspase-3 and Caspase-9) in the CC group. Remarkably, following treatment with 9B and 12B, the expression of IL-6, STAT-3, and Bcl-2 decreased, whereas the expression of caspases increased. Bax and Cyt-C also showed the higher expression with 12B treatment than with 9B treatment in the CC group (Fig. 6).

## Discussion

The most prevalent and deadly solid cancer is LC due to uncontrolled respiratory exponentiation [30]. 5-FU is a well-known anti-cancer drug which consisting a thiazole, and a pyrimidine ring, and is considered as standard for the treatment of different solid tumors. Therefore, we synthesized a new series of synthetic molecules with thiazolo[3,2-a]pyrimidine ring by adopting rational drug design strategies [16]. All synthesized thiazolo[3,2-a]pyrimidine analogues were screened against A549 cell line for their cytotoxic potential. In the series of 15 compounds, 9B and 12B exhibited the best cytotoxic effect in the SRB assay among the entire synthesized compounds against the A549 cell line. Similar study conduted by *keshari et al.*, showed cytotoxic potential of the thiazolo[3,2-a]pyrimidine analogues against Hep-G2 cell line. In addition, a molecular docking study showed a good binding affinity and better stability with IL-6, Cyt-C, caspase-9 and caspase-3 [27–31] as well as hydrogen bondings with both compounds. Moreover, 9B and 12B compounds have the strong binding affinity with the caspase 9 (-7.5 kcal/mol and − 7.3 kcal/mol) and caspase 3 (-7.4 kcal/mol and − 7.2 kcal/mol) respectively. Hydrogen bonds involvement with caspases suggesting strong binding activity with them. Caspases play an important role in initiating and executing apoptosis [32, 33]. Accordingly, it might be a promising antiproliferative activity containing compounds.

The acute toxicity studies of thiazolo[3,2-a]pyrimidines containing 9B and 12B compounds showed no toxic effect in the histological and oxidative scrutinization at 5, 10, and 15 mg/kg dose. Therefore, we selected a dose of 10 mg/kg (p.o.) for further assessments similar to 5-FU [10, 11, 13].

The pharmacokinetic studies demonstrated a greater plasma volume distribution of 9B and 12B, with 12B demonstrating better oral bioavailability than 9B. From the findings of hydrogen bond donors, the molecular weight and binding affinity of both 9B and 12B compounds can be further used as good orally absorbed compounds [16].

The significant cytotoxic potential, binding affinity, safety profile, and oral bioavailability of 9B and 12B paved the way for further *in-vivo* estimations against EC-induced LC in albino Wistar rats. Higher weight loss in the CC group was observed because of cancer cachexia and loss of adipose tissue [34, 35]. At a dose of 10 mg/kg, 12B demonstrated better efficacy in weight restoration than 9B.

Various empirical research has indicated that changes in lipid metabolism are considered to be a characteristic feature of carcinogenesis [36]. Cancer cells required more energy for their proliferation by *denovo* reactivated lipid synthesis. Additionally, overexpression of multifunctional enzyme fatty acid synthases, play key role in poor prognosis of LC due to significantly increased cancer aggressiveness [37]. Several studies proven that LDL concentration is a prognostic factor in small cell lung cancer. High TG concentration and lower HDL created a predisposition to higher LC incidence [36, 38]. Additionally, Increased levels of TC and LDL are connected with the cancer growth [39] and potentially associated with increased cellular proliferation [29, 40]. On the other hand, HDL involvement in the reverse transport process of cholesterol facilitates the removal of excess cholesterol levels from peripheral tissues [41]. Additionally, an inverse relationship between cancer and HDL was demonstrated in LC. Several studies have shown that increased HDL levels may be associated with anti-inflammatory effects through cytokine protection and leukocyte adhesion; therefore decreased HDL may reduce inflammation and antioxidant activity as well as cancer development [42, 43]. Our results showing TC, TG, and LDL level in EC-treated animals are consistent with the aforementioned research.

Previous studies have reported that EC is metabolized to vinyl carbamate, followed by the formation of epoxide, which interacts with nucleic acids to trigger ROS formation and DNA damage [44]. The resultant ROS generation and DNA damage lay the foundation for the development and progression of cancer [44], which was evident with increased levels of ProC and TBARs in the CC group. Concomitant administration of 9B and 12B resulted in decreased levels of ProC and TBARs, which could be attributed to the presence of electronegative substitution available on the thiazolo[3,2-a]pyrimidine ring [45]. The antioxidant potential of 9B and 12B was further affirmed through the restoration of the enzymatic activity of SOD and GSH. The free radical scavenging potential of 9B and 12B can be attributed to the thiazolo[3,2-a]pyrimidine ring, which is consistent with previous reports [8].

The efficacy of 9B and 12B was further scrutinized through pro-inflammatory markers (IL-2, IL-6, IL-10, and IL-1β). EC administration was evident in the upsurge in the levels of inflammatory cytokines [35, 46, 47]. These findings suggest that EC-induces inflammatory signaling associated with anti-apoptotic mechanisms, which is corroborated by previous reports [34].

Histopathological analysis of CC revealed the loss of tissue architecture, alveolar damage, elevated mucinous adenocarcinoma cells, and inflammatory cells to support the proliferation of the lung alveolar epithelium. However, treatment with 9B and 12B showed a healthy architecture of the bronchioles, and alveoli, reduced lung proliferation and restoration of tissue architecture.

In morphological analysis of intact organs, the existence of tumor formation was higher in the EC-induce model, which was further reduce after treatment with 9B and 12B. SEM analysis revealed the presence of a capillary network interlining the fibrous structure of the alveolar wall in NC [34]. However, the CC group accounted for the parenchymal compaction with reducing the size of annuli and loss of preferred region [48]. The 12B treated group demonstrated better efficacy than 9B for restoration of tissue architectures.

The administration of 9B and 12B curbed inflammatory markers and restored the apoptotic machinery, evident through the upregulation of caspase-3 and caspase-9 levels. The findings reported above clearly demonstrate the anticancer potential of 9B and 12B against EC-induced LC in albino Wistar rats. However, to investigate the possible mechanism underlying the biological activity of 9B and 12B, we scrutinized the expression of various apoptotic markers using immunoblotting.

Pro-inflammatory IL-6 regulates STAT3 to promote Bcl-2 activation and prevent Cyt-C release [49, 50]. The release of Cyt-C is an important step in the intrinsic apoptotic pathway, where caspase 3 and 9 are key regulators [51]. In contrast, caspase-9 is a key step in intrinsic apoptosis [51]. In view of the above, we examined the expression of IL-6, STAT3, Bcl-2, Bax, Cyt-C, caspase-3, and caspase-9 in lung tissue samples.

In the CC group, we observed increased expression of IL-6, STAT3, and Bcl-2 with decreased expression of Cyt-C, caspase-9, and caspase-3. IL-6 is a potent cytokine that regulates the immune defense process of the body. IL-6 is a crucial link between inflammation and cancer. Higher IL-6 concentrations are indicated by secretion through tumor-associated macrophages or tumor cells due to malignancy-induced chronic stress. IL-6 release involves different tyrosine kinase pathways or transcription 3 activation. STAT3 deficiency indicates tumor growth, and IL-6/STAT3 has a crucial role in carcinogenesis [52]. Oral administration of 9B and 12B significantly downregulates IL-6, STAT3, and Bcl-2 expression, indicating the initiation of the apoptotic mechanism. Previous studies have implying a family of cysteine proteases, formally known as caspases, is associated with the process of apoptosis induction [53]. In the continuation of this study, 9B and 12B upregulate the expression of Cyt-C, caspase-9, and caspase-3, which affirms that both the analogues exert anticancer activity against LC by regulating the intrinsic apoptotic mechanism. This could serve as potential lead molecules for the development of LC drugs in the future for clinical and therapeutic research.

## Conclusion

The present study sheds light on the ameliorative effect of indeno[1,2-d]thiazolo[3,2-a]pyrimidine analogues. *In-vitro, in-silico*, and *in-vivo* studies showed the antiproliferative effects of both synthesized compounds. 9B and 12B showed significant antiproliferative activity against A549 LC cells. Molecular docking, morphological, biochemical, anti-inflammatory, and antioxidant profiling, as well as the expression of apoptotic proteins, all supported that 9B and 12B could control the growth of EC-induced LC. Based on the overall findings, we can speculate that both synthetic compounds may be considered novel therapeutic compounds for LC treatments.

### Electronic supplementary material

Below is the link to the electronic supplementary material.

Supplementary Material 1

## Data Availability

The datasets generated or analyzed in this study are accessible upon reasonable request..

## Abbreviations

5-FU: 5-Fluorouracil
A549: Adenocarcinomic human alveolar basal epithelial cells
AD: Adenocarcinoma
ADR: Adriamycin
AUC: Area under the curve
BAX: Bcl-2-associated X protein
Bcl-2: B-cell lymphoma 2
CMC: Carboxymethyl cellulose
CYP2E1: Cytochrome P450 2E1
CYP450: Cytochrome P450
Cyt-C: Cytochrome complex
DNA: Deoxyribonucleic acid
EC: Ethyl Carbamate or Urethane
ECL: Enhanced chemiluminescent
ELISA: Enzyme-linked immunosorbent assay
GSH: Glutathione
H&E: Hematoxylin and eosin
HepG-2: Hepatoblastoma cell line
HPLC: High-performance liquid chromatography
HSV-1: Anti-herpes simplex virus type-1
IARC: International Agency for Research on Cancer
LC: Lung cancer
LCC: Large cell carcinoma
Mcl-1: Myeloid cell leukemia sequence 1
NSCLC: Non-small-cell lung cancer
ProC: Protein-Carbonyl
PVDF: Polyvinylidene fluoride
RIPA: Radio Immunoprecipitation Assay
SCC: Squamous cell carcinoma
SCLC: Small cell lung cancer
SOD: Superoxide Dismutase
SRB assay: Sulforhodamine B assay
STAT3: Signal transducer and activator of transcription 3
TBARS: Thiobarbituric acid reactive substances
TBS-T: Tris-buffered Tween-20
WB: Western Blot
