# Supplementary material for: Antiproliferative effect of indeno[1,2-d]thiazolo[3,2-a]pyrimidine analogues on IL-6 mediated STAT3 and role of the apoptotic pathway in albino Wistar rats of ethyl carbamate-induced lung carcinoma: In-silico, In-vitro, and In-vivo study
Source: Cancer Cell Int. 2024 Jun 26;24:219. doi: 10.1186/s12935-024-03390-6 (PMC11201866; doi:10.1186/s12935-024-03390-6)
Supplement: Supplementary file 1 — Supplementary Material 1 [file 12935_2024_3390_MOESM1_ESM.docx]

**Table 1** The various substitutions and *in-vitro* cytotoxicity data of synthesized derivatives against Human Lung Cancer Cell Line A-549 (1B-15B)

| **Comp. Code** | **X** | **R_1_** | **R_2_** | **R_3_** | **R_4_** | **Y_1_** | **Y_2_** | **GI_50_**  **µg/mL** | **LC_50,_**  **µg/mL** | **TGI**  **µg/mL** |
| --- | --- | --- | --- | --- | --- | --- | --- | --- | --- | --- |
| 1B | -H | -H | -H | -Br | -H | -H | -H | >80 | NE | NE |
| 2B | -H | -Cl | -H | -H | -H | -H | -H | >80 | NE | NE |
| 3B | -H | -H | -H | -Br | -H | -H | -CH_3_ | >80 | NE | NE |
| 4B | -OCH_3_ | -H | -H | -Br | -H | -CH_3_ | -H | >80 | NE | NE |
| 5B | -H | -H | -H | -Br | -H | -CH_3_ | -H | >80 | >80 | NE |
| 6B | -OCH_3_ | -H | -H | -Br | -H | -H | -H | >80 | NE | NE |
| 7B | -OCH_3_ | -H | -H | -Br | -H | -H | -CH_3_ | >80 | NE | NE |
| 8B | -H | -H | -OCH_3_ | -OCH_3_ | -OCH_3_ | -H | -H | >80 | NE | NE |
| 9B | -OCH_3_ | -H | -OCH_3_ | -OCH_3_ | -OCH_3_ | -H | -H | **<20** | >80 | NE |
| 10B | -H | -H | -H | -Cl | -H | -CH_3_ | -H | >80 | NE | NE |
| 11B | -OCH_3_ | -H | -H | -Cl | -H | -CH_3_ | -H | >80 | NE | NE |
| 12B | -H | -H | -H | -Cl | -H | -H | -H | **<10** | NE | NE |
| 13B | -OCH_3_ | -H | -H | -Cl | -H | -H | -H | >80 | NE | NE |
| 14B | -H | -H | -H | -Cl | -H | -H | -CH_3_ | >80 | >35.6 | NE |
| 15B | -OCH_3_ | -H | -H | -Cl | -H | -H | -CH_3_ | >80 | NE | NE |
| **ADR** |  |  |  |  |  |  |  | **<10** | NE | <10 |

LC_50_ = Concentration of drug causing 50% cell kill

TGI = Concentration of drug causing total inhibition of cell growth

GI_50_ = Concentration of drug causing 50% inhibition of cell growth

ADR = Adriamycin, Positive control compound

**NE** stands for Non-evaluable data. The experiment needs to be repeated using a different set of drug concentrations

**Supplementary data**

**Comment 2:**

As per reviewer suggestion 2, we performed the docking study with EGFR (Cancer signaling pathway) to review.

**Figure .** Binding affinities of compound 9B and 12B. **(I)** 3D and 2D molecular docking images of 9B (1 and 1’) VGFR-2 (PDB: 3EWH) and (2 and 2’) Caspase 8 (PDB: 3KJN). and **(II)** 3D and 2D docking images of 12B (3 and 3’) VGFR-2 (PDB: 3EWH) and (4 and 4’) Caspase 8 (PDB: 3KJN). Comparative studies were performed using AUTODOCK 1.5.6.

**
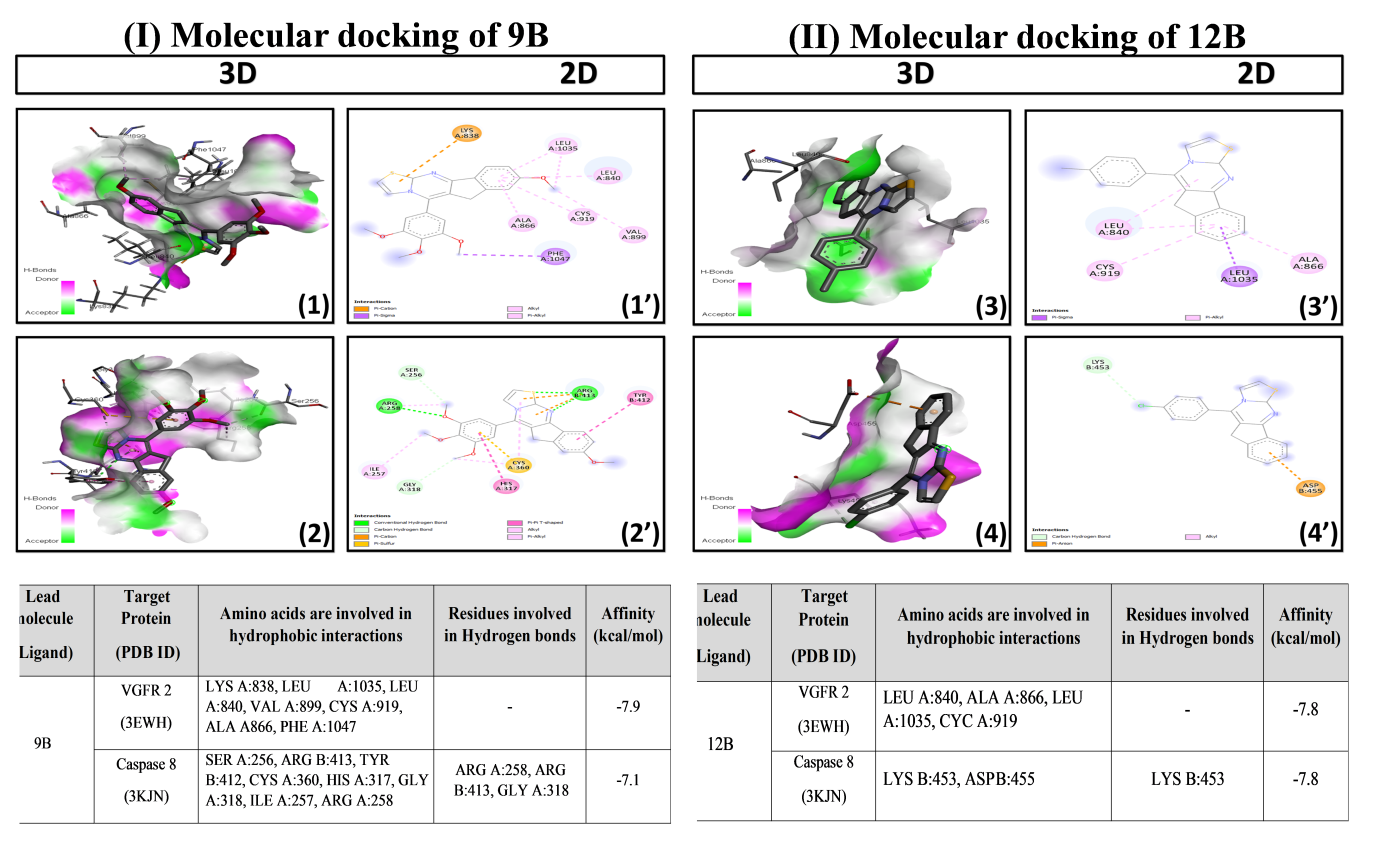
**

**Comment 3:**

As per comment 3, we have included a one-pot efficient synthetic procedure, physicochemical properties, and spectral characterizations of synthesized compounds (9B and 12B) for your review.

**One-pot efficient synthesis of substituted indeno[1,2-d]thiazolo[3,2-a]pyrimidines (1B–15B):**

A mixture of substituted α-indanone (1 mmol), appropriate aromatic aldehydes (1 mmol), and distinctive 2-aminothiazoles (1 mmol) in EtOH (5.0 mL) in the presence of 20 mol% p-TSA was heated under reflux for 3–4 h. The reaction mixture was then poured into ice-cold water. A solid product was obtained, which was filtered, washed thoroughly with distilled water, and recrystallized from EtOH. Pure crystals were obtained **(Scheme 1).** The progress of the reaction was monitored by TLC on precoated silica gel-G plates using 30% ethylacetate:n-hexane as the solvent system. TLC revealed just a single spot, which proved the presence of a single product.


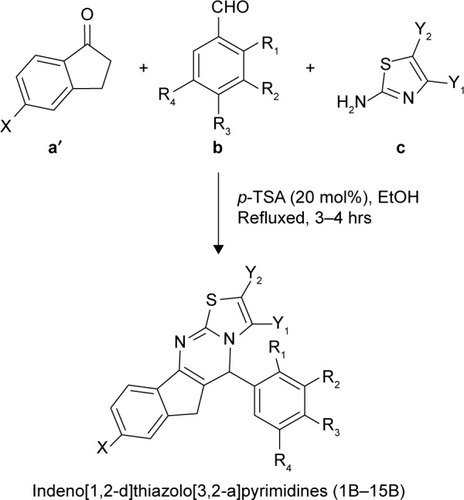


**Scheme 1:** **One-pot efficient synthetic route to the titled compounds (1B–15B)**

**Note**: Substituted α-indanone (**a′**), substituted aromatic aldehydes (**b**) and distinctive 2-aminothiazoles (**c**).
**Abbreviations**: EtOH, ethanol; *p*-TSA, *p*-toluenesulfonic acid.

**Various substitutions of the synthesized derivatives (9B and 12B):**

**8-methoxy-5-(3,4,5-trimethoxyphenyl)-5,6-dihydroindeno[1,2-d]thiazolo[3,2-a]pyrimidine (9B): Mol. wt. 422.50**

**5-(4-chlorophenyl)-5,6-dihydroindeno[1,2-d]thiazolo[3,2-a]pyrimidine (12B): Mol. wt. 336.84**

| **Comp. code** | **X** | **R1** | **R2** | **R3** | **R4** | **Y1** | **Y2** |
| --- | --- | --- | --- | --- | --- | --- | --- |
| **9B** | –OCH3 | –H | –OCH3 | –OCH3 | –OCH3 | –H | –H |
| **12B** | –H | –H | –H | –Cl | –H | –H | –H |

**Table : Various substitutions of the synthesized derivatives (9B & 12B):**

**Physicochemical Properties and Spectral Characterizations of Synthesized Compounds (9B & 12B):**

**8-methoxy-5-(3,4,5-trimethoxyphenyl)-5,6-dihydroindeno[1,2-d]thiazolo[3,2-a]pyrimidine (9B)**: Yellow crystals; yield: 66%; m.p. 208°C–210°C; Rf=0.68 (SiO2, ethylacetate : n-hexane, 3:7, v/v). **^1^H NMR** (DMSO-d6, 800 MHz) δ ppm: 3.35 (s, 3H), 3.74 (s, 6H), 3.91 (s, 4H), 4.15 (s, 2H), 7.04 (dd, 1H, J=8 Hz and J=8 Hz), 7.10 (s, 3H), 7.23 (s, 1H), 7.44 (s, 1H), 7.73 (d, 1H, J=8 Hz). **^13^C NMR** (DMSO-d6, 200 MHz) δ ppm: 32.22, 39.76, 39.86, 39.97, 40.07, 40.18, 56.31, 56.44, 60.63, 108.59, 110.41, 116.15, 125.87, 131.03, 132.40, 135.15, 139.32, 153.49, 165.38, 192.08. **LCMS (m/z):** 421.15 [M−H]^-,^ prominent fragment peaks were observed at 375.10 [M - OCH₃, trimethoxy group]⁺, 255.25 [M - C₉H_11_O₃]⁺ corresponding to the loss of specific functional groups and structural segments.

**5-(4-chlorophenyl)-5,6-dihydroindeno[1,2-d]thiazolo[3,2-a]pyrimidine (12B):** Yellow crystals; yield: 65%; m.p. 176°C–178°C; Rf=0.51 (SiO2 , ethylacetate : n-hexane, 3:7, v/v). **^1^H NMR** (DMSO-d6, 800 MHz) δ ppm: 4.13 (s, 3H), 7.50(s, 1H), 7.54 (s, 1H), 7.57 (d, 2H, J=8 Hz), 7.68 (d, 1H, J=8 Hz), 7.72 (d, 1H, J=8 Hz), 7.80–7.83 (m, 4H). **^13^C NMR** (DMSO-d6, 200 MHz) δ ppm: 32.28, 39.75, 39.86, 39.96, 40.07, 40.17, 124.17, 127.19, 128.26, 129.51, 131.91, 132.88, 134.28, 134.93, 135.55, 136.25, 137.55, 150.52, 193.77. **LCMS (m/z):** 339.15 [M+2].
